# Molecular Epidemiology of Human Adenovirus–Associated Febrile Respiratory Illness in Soldiers, South Korea^1^

**DOI:** 10.3201/eid2407.171222

**Published:** 2018-07

**Authors:** Jung Yeon Heo, Ji Yun Noh, Hye Won Jeong, Kang-Won Choe, Joon Young Song, Woo Joo Kim, Hee Jin Cheong

**Affiliations:** Ajou University School of Medicine, Suwon, South Korea (J.Y. Heo);; Korea University College of Medicine, Seoul, South Korea (J.Y. Noh, J.Y. Song, W.J. Kim, H.J. Cheong);; Chungbuk National University College of Medicine, Cheongju, South Korea (H.W. Jeong);; The Armed Forces Capital Hospital, Seongnam, South Korea (K.-W. Choe)

**Keywords:** adenovirus infections, human, HAdV, soldiers, military personnel, respiratory tract infections, acute febrile respiratory illness, AFRI, viruses, South Korea

## Abstract

During January 2013–April 2014, we subjected nasopharyngeal specimens collected from patients with acute febrile respiratory illness in a military hospital to PCR testing to detect 12 respiratory viruses and sequence a partial hexon gene for human adenovirus (HAdV) molecular typing. We analyzed the epidemiologic characteristics of HAdV infections and compared clinical characteristics of HAdV types. Among the 305 patients with acute febrile respiratory illness, we detected respiratory viruses in 139 (45.6%) patients; HAdV was the most prevalent virus (69 cases). Of the 40 adenoviruses identified based on type, HAdV-55 (29 cases) was the most prevalent, followed by HAdV-4 (9 cases). HAdV-55 was common in patients with pneumonia (odds ratio 2.17; 95% CI 0.48–9.86) and hospitalized patients (odds ratio 5.21; 95% CI 1.06–25.50). In soldiers with HAdV infection in Korea, HAdV-55 was the most prevalent type and might be associated with severe clinical outcomes.

Human adenoviruses (HAdVs) are considered the most important causative agent of acute respiratory infection in soldiers, particularly in new recruits (*1**,**2*). A total of 79 HAdV types have been documented (*3*). The distribution of HAdV types differs substantially by geographic region and environmental factors (*4**,**5*). HAdVs are prevalent in training facilities or military barracks for soldiers, but the prevalent types of HAdV have changed over time (*6*). Historically, HAdV-4 and HAdV-7 have been the most prevalent causes of acute febrile respiratory illness (AFRI) among US military personnel since the 1950s (*7*). Vaccination against HAdV-4 and HAdV-7 has been effective in reducing AFRI among US military trainees to date (*8**,**9*). However, since 2007, the emergence of new adenovirus types such as HAdV-14, which is distinct from the prototype in the United States, has been associated with outbreaks of AFRI and severe pneumonia (including several deaths) in military populations (*10**,**11*).

HAdV is most prevalent in patients with acute lower respiratory tract infection and is the most common cause of pneumonia among military personnel in South Korea (*12*). However, studies evaluating the types of HAdV in this population are limited because of the lack of knowledge about HAdVs among military physicians. In 2012, HAdV-55 was identified in patients with severe pneumonia, and an outbreak of AFRI among military personnel in South Korea was recorded (*13**,**14*). HAdV-55, which is a novel HAdV type characterized by genome recombination between HAdV-B11 and HAdV-B14, caused outbreaks of acute respiratory diseases in military camps in Turkey, China, and Singapore (*15**–**18*). Although cases of pneumonia caused by HAdV-55 among military personnel in South Korea have been recorded, information on the epidemiology and characteristics of type-specific HAdV respiratory infections among military personnel in South Korea is limited. Thus, our study aimed to investigate the epidemiology of HAdV infections and to compare the clinical characteristics by type of HAdVs in soldiers in South Korea via hospital-based surveillance on viral respiratory infections.

## Methods

### Characteristics of the Study Population and Case Definition

The study was approved by the Institutional Review Board of the South Korea Armed Forces Medical Command. During January 2013–April 2014, we enrolled in the study all new recruits and active duty soldiers with AFRI who were required to visit the emergency department or to undergo hospitalization at the Armed Forces Capital Hospital in Seongnam, South Korea. We defined AFRI as a history of fever or measured fever >37.6°C and the presence of >1 respiratory symptom such as cough, sore throat, or rhinorrhea with onset within the last 7 days. The Armed Forces Capital Hospital is the only tertiary care hospital in the South Korea military healthcare system. Furthermore, the hospital offers primary and secondary medical services to soldiers in the city of Seoul and Gyeonggi Province. Almost all soldiers in South Korea, except officers, use military hospitals for free health services.

### Respiratory Virus Multiplex Reverse Transcription PCR

We collected nasopharyngeal or throat swab specimens from the patients with AFRI within 24 hours after their hospital visit by using a flocked swab. We stored the specimen at 4°C in viral transport media until further testing. Within 3 days of collection, we sent specimens to a commercial laboratory center (GC Labs, Yongin, South Korea), where they were subjected to respiratory virus multiplex reverse transcription PCR.

We extracted total viral nucleic acid from the specimens by using the Chemagic Viral DNA/RNA Extraction Kit (Chemagen Inc., Baesweiler, Germany) and performed cDNA synthesis by using the CapFishing Full-Length cDNA Premix Kit (Seegene Inc., Seoul, South Korea). We performed PCR by using the Seeplex RV12 ACE Detection Kit (Seegene Inc., South Korea), which is used for identifying influenza viruses A and B, respiratory syncytial viruses A and B, adenovirus, parainfluenza virus types 1–3, rhinovirus group A, human coronavirus 229E/NL63, human coronavirus OC43, and human metapneumovirus.

### Molecular Analysis of HAdVs

We extracted DNA from the adenovirus-positive respiratory specimens by using the QIAamp DNA Blood Mini Kit (QIAGEN, Hilden, Germany). We amplified the partial nucleotides of the hexon gene by using PCR as described elsewhere with some modifications (*19*). We amplified viral sequences by using oligonucleotide primers producing a 475-bp fragment: ADHEX1F (5′-CAACACCTAYGASTACATGAA-3′) and ADHEX1R (5′-KATGGGGTARAGCATGTT-3′). PCR conditions were as follows: initial denaturation at 94°C for 1 min, followed by 35 cycles of denaturation at 94°C for 1 min, annealing at 50°C for 1 min, elongation at 68°C for 1 min, and final extension at 68°C for 5 min. For samples that tested negative in the first PCR reaction, we performed heminested PCR by using primers (273 bp) ADHEX1F (5′-CAACACCTAYGASTACATGAA-3′) and ADHEX2R (5′-ACATCCTTBCKGAAGTTCCA-3′) with the same temperature and time profiles. We determined DNA sequences in both directions by using the Applied Biosystems Automatic Sequencer ABI 3730xl and ABI Prism BigDye Terminator v3.1 sequencing system (Applied Biosystems, Foster City, CA, USA). We identified the type of HAdV by using BLAST (http://blast.ncbi.nlm.nih.gov/Blast.cgi).

We generated phylogeny on the basis of the 232-bp nucleotide sequences of the hexon gene of HAdVs. For the phylogenetic analysis, we selected the sequences of each type of HAdV from GenBank. We used MEGA 6 software to generate the phylogenetic tree and evaluated topologies by performing a bootstrap analysis of 1,000 iterations (*20*).

### Collection of Clinical Data

We obtained clinical information of the patients with HAdV respiratory infection during the study period from standardized case report forms. The case report forms, which included clinical diagnosis, intensive care unit stay, requirement for mechanical ventilation or vasopressor, and symptoms at presentation, were completed within 7 days of the hospital visit by an attending physician.

### Statistical Analysis

We conducted Pearson’s χ^2^ and Fisher exact test for the demographic and clinical variables by using the SPSS for Windows version 20 (IBM Corp., Armonk, NY, USA). For all analyses, we defined statistical significance as p<0.05.

## Results

### Epidemiology of HAdVs in Soldiers with AFRI

During January 2013–April 2014, we enrolled 305 patients with AFRI in the study. We detected a total of 157 respiratory viruses in 139 (45.6%) soldiers with AFRI. HAdV was the most prevalent virus (49.6% [69/139]), followed by influenza A or B virus (28.8% [40/139]) and rhinovirus group A (12.2% [17/139]). Among 139 cases in which respiratory viruses were identified, >2 viruses were detected in 18 cases (12.9%) (Table 1).

**Table 1:** Respiratory viruses in soldiers with acute febrile respiratory illness, South Korea, January 2013–April 2014

Respiratory virus	No. (%) with virus identified*	No. (%) with >2 viruses identified
Adenovirus	69 (49.6)	12 (17.4)
Influenza A or B	40 (28.8)	3 (7.5)
Rhinovirus group A	17 (12.2)	6 (35.3)
Coronavirus 229E/NL63 or OC43	11 (7.7)	5 (45.4)
Respiratory syncytial virus A or B	10 (7.9)	4 (40.0)
Parainfluenza virus 1, 2, or 3	9 (6.5)	5 (55.6)
Metapneumovirus	1 (0.7)	1 (100.0)

*Among the 139 soldiers in whom a respiratory virus was detected.

**Figure 1:**
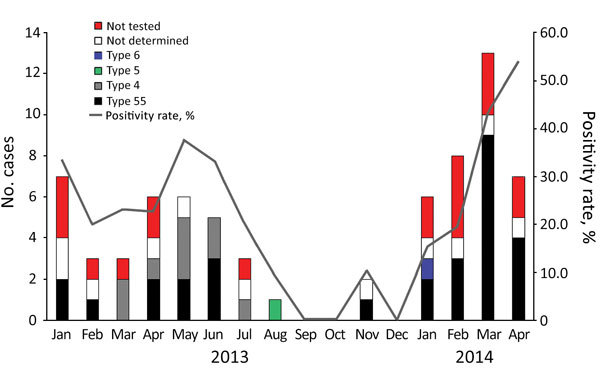
Temporal distribution of acute febrile respiratory illness from human adenovirus (HAdV) infection among soldiers (no. cases) and overall HAdV positivity rate among collected specimens, by HAdV type, South Korea, January 2013–April 2014. We observed HAdV respiratory infection primarily during winter and spring. In 2014, acute febrile respiratory illness in soldiers in South Korea was almost always associated with HAdV-55. Co-circulation of HAdV-55 and HAdV-4 occurred during spring and early summer of 2013.

We commonly observed cases of HAdV infection during winter and spring (Figure 1). Among the tested cases of AFRI, the monthly positive rate for HAdV ranged from 0% to 53.8%; average positivity rate was 22.6%. A peak positive rate for HAdV infection occurred in soldiers with AFRI during March–April 2014. The mean age of the patients with HAdV infection was 21.7 years. Among the 69 patients with HAdV infection, 40.6% were new recruits and 75.4% were hospitalized (Table 2).

**Table 2:** Demographic characteristics of soldiers with HAdV respiratory infection, by HAdV type, South Korea, January 2013–April 2014*

Characteristic	HAdV type

Type 55	Type 4	Type 5	Type 6	Not determined	Unable to type	Total
No. (%) cases	29 (42.0)	9 (13.0)	1 (1.4)	1 (1.4)	11 (15.9)	18 (26.1)	69 (100.0)
Age, y, mean +SD	21.41 +1.92	21.33 +1.32	21	20	21.42 +1.75	21.63 +1.38	21.69 +1.49
Military rank, no. (%)							
New recruits	12 (41.4)	3 (33.3)	1 (100.0)	0	5 (45.5)	7 (38.9)	28 (40.6)
Active-duty soldiers	17 (58.6)	6 (66.7)	0	1 (100.0)	7 (63.6)	10 (55.6)	41 (59.4)
Smokers, no. (%)	15 (51.7)	5 (55.6)	0	0	7 (63.6)	11 (61.1)	38 (55.1)
Hospitalized, no. (%)	25 (86.2)	6 (66.7)	0	0	8 (72.7)	13 (72.2)	52 (75.4)

*All soldiers were male. Some HAdV types were not verified despite our performing molecular analyses. Molecular analyses for typing of HAdV type were not performed. HAdV, human adenovirus.

**Figure 2:**
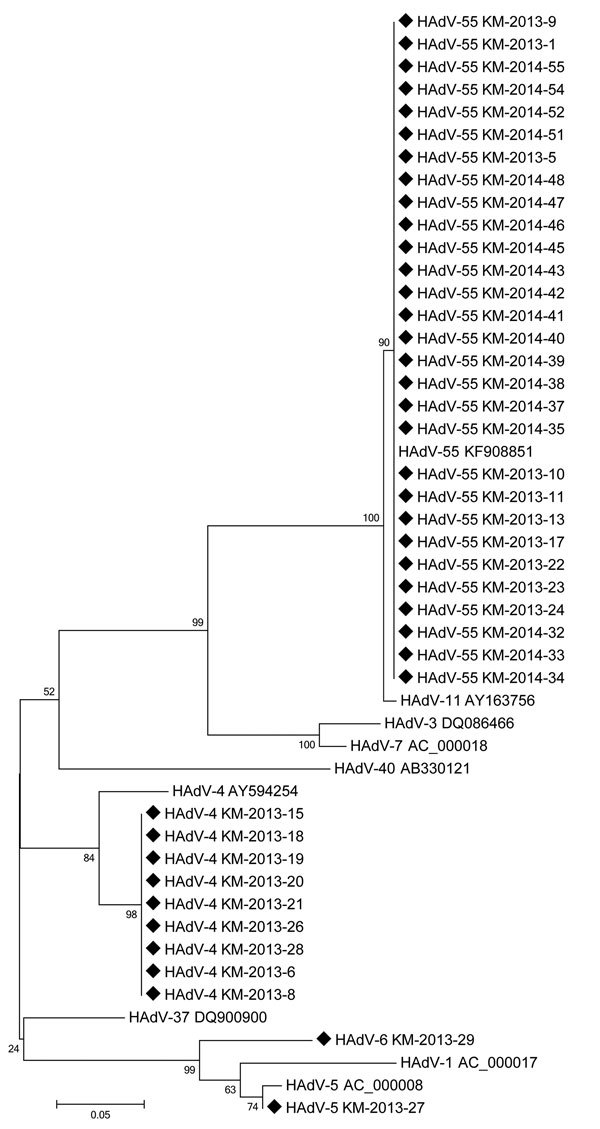
Phylogenetic tree human adenoviruses detected among soldiers with acute febrile respiratory illness from human adenovirus (HAdV) infection, South Korea, January 2013–April 2014. Tree was constructed by the neighbor-joining method on the basis of a 232-bp nucleotide sequence of the hexon gene. We used MEGA 6 software (*20*) to generate the phylogenetic tree and evaluated topologies by using bootstrap analysis of 1,000 iterations. GenBank accession numbers of sequences of HAdV from the soldiers with acute febrile respiratory illness (indicated by black diamonds) were KX227462–KX227469 and KX513954–KX513985. GenBank accession numbers of reference adenovirus sequences are shown in the tree. Scale bar indicates nucleotide substitutions per site. HAdV, human adenovirus.

From the 69 patients with HAdV infection, 51 respiratory specimens were available for further molecular analysis. For the 51 samples tested, we identified the HAdV type in 40 samples (Figure 2). HAdV-55 (72.5% [29/40]) was the most prevalent type in soldiers with HAdV infection, followed by HAdV-4 (22.5% [9/40]). We detected HAdV-55 and HAdV-4 in 2013, but we did not detect HAdV-4 in 2014. We detected HAdV-5 and HAdV-6 in 1 case each.

### Clinical Characteristics of HAdV-55 Infection versus Other Types of HAdV Infections

We observed no statistically significant difference in the demographic characteristics or signs and symptoms of the patients with HAdV-55 infection compared with those with other types of HAdV infection (Table 3). Furthermore, we observed no statistically significant difference between the patients in terms of laboratory and radiographic findings. Co-infections with other bacteria or viruses were similar in both groups. The patients with HAdV-55 infection were more likely to have onset pneumonia (44.8% vs. 27.3% [odds ratio (OR) 2.17; 95% CI 0.48–9.86]) and be hospitalized (86.2% vs. 54.5% [OR 5.21; 95% CI 1.06–25.50]) than those infected with other types of HAdV. In particular, we identified HAdV-55 infection in all patients who required hospitalization in the intensive care unit or mechanical ventilation caused by acute respiratory distress syndrome (ARDS).

**Table 3:** Comparison of demographic and clinical features of soldiers with acute febrile respiratory illness, by HAdV type 55 infection status, South Korea, January 2013–April 2014*

Characteristic	HAdV-55 infection status		OR (95% CI)	p value
	HAdV-55, n = 29	Non–HAdV-55, n = 11		

Age, y, mean +SD	21.41 +1.92	21.18 +1.40	NA	0.717†
New recruits	12 (41.4)	5 (45.5)	0.84 (0.21–3.43)	0.816‡
Signs and symptoms at presentation				
Fever >5 d	17 (58.6)	5 (45.5)	1.70 (0.42–6.88)	0.455‡
Nausea/vomiting	5 (17.2)	2 (18.2)	0.93 (0.15–5.73)	1.000§
Diarrhea	6 (20.7)	3 (27.3)	0.70 (0.14–3.45)	0.686§
Dyspnea/tachypnea	8 (27.6)	1 (9.1)	3.81 (0.42–34.8)	0.399§
Conjunctival injection	4 (13.8)	2 (18.2)	0.72 (0.11–4.63)	1.000§
Laboratory findings, mean +SD				
Leukocyte count, cells/μL	5,200 +1,818	6,322 +2,656	NA	0.134†
Platelet count, × 10^3^ cells/μL	177 +57	172 +77	NA	0.848†
C-reactive protein, mg/dL	6.7 +3.9	5.0 +1.8	NA	0.193†
Radiographic findings				
Bilateral involvement	6 (20.7)	1 (9.1)	2.61 (0.28–24.59)	0.650§
Consolidation	11 (37.9)	2 (18.2)	2.75 (0.50–15.14)	0.286§
Patchy infiltration	3 (10.3)	1 (9.1)	1.15 (0.11–12.44)	1.000§
Effusion	3 (10.3)	1 (9.1)	1.15 (0.11–12.44)	1.000§
Clinical diagnosis				
Pharyngitis	12 (41.4)	5 (45.5)	0.85 (0.21–3.43)	0.815‡
Tracheobronchitis	4 (13.8)	3 (27.3)	0.43 (0.08–2.33)	0.369§
Pneumonia	13 (44.8)	3 (27.3)	2.17 (0.48–9.86)	0.473§
Co-identified bacteria	2 (6.9)	1 (9.1)	0.74 (0.06–9.09)	1.000§
* Streptococcus pneumoniae*	1 (3.4)	1 (9.1)	–	–
* Mycoplasma pneumoniae*	1 (3.4)	0	–	–
Co-identified viruses	5 (17.2)	1 (9.1)	2.08 (0.22–20.17)	1.000§
Rhinovirus	3 (10.3)	0	–	–
Coronavirus	1 (3.4)	1 (9.1)	–	–
Parainfluenza virus	1 (3.4)	0	–	–
Metapneumovirus	1 (3.4)	0	–	–
Hospitalized patients	25 (86.2)	6 (54.5)	5.21 (1.06–25.50)	0.083§
Admission to the intensive care unit	7 (24.1)	0	NA	0.159§
Acute respiratory distress syndrome	5 (17.2)	0	NA	0.298§
Length of hospital stay, d, mean +SD	16.2 +9.9	14.6 +5.6	NA	0.619†
Death	0	0	NA	NA

*Values are no. (%) soldiers except as indicated. HAdV, human adenovirus; NA, not available; OR, odds ratio; –, not applicable. †By Student *t*-test. ‡By χ^2^ test. §By Fisher exact test.

## Discussion

In our study, HAdV was the most prevalent virus detected among soldiers with AFRI in South Korea, representing 49.6% of the cases. HAdV-55 was the most prevalent type among the cases that could be identified using PCR. Although HAdV-55 has recently received public attention as an emerging pathogen that causes outbreaks of respiratory illness and severe pneumonia in the general population and soldiers, acute respiratory illness associated with HAdV-55 has rarely been reported in the civilian population in South Korea (*6**,**16**,**21**–**23*). In children in South Korea, HAdV-3 and HAdV-7 are prevalent serotypes and have also been associated with the outbreaks of adenoviral respiratory illness since the 1990s (*24**,**25*). Severe pneumonia cases associated with HAdV-55 infection were recently reported in soldiers in South Korea (*13**,**14*). Our finding that HAdV-55 was the most prevalent type in soldiers in South Korea differs from the epidemiology of adenovirus in children in South Korea.

Another notable finding of this study was the changing epidemiologic trend from the co-circulation of HAdV-4 and HAdV-55 in 2013 to the predominant circulation of HAdV-55 in 2014. HAdV-4 was the second most common type after HAdV-55 among soldiers with AFRI in South Korea in 2013. However, HAdV-4 has not been identified in soldiers in South Korea since November 2013. Although the shift in the HAdV types in soldiers in South Korea could not be fully understood because of the relatively short study period, HAdV-55 infection has been prevalent among soldiers in South Korea since 2014 (*14**,**26*).

Pneumonia and hospitalization associated with HAdV-55 infection were more frequent than those associated with the other types of HAdV infection in soldiers in South Korea. In particular, HAdV-55 infection was associated with severe pneumonia or ARDS. Nevertheless, we observed no significant differences between HAdV-55 cases and non–HAdV-55 cases in terms of the frequency of clinical diagnosis of pneumonia, hospitalization, and ARDS. These findings could be explained by 2 assumptions. First, specific HAdV types such as HAdV-3, HAdV-7, or HAdV-14 might be more virulent than other types (*25**,**27**,**28*). Considering that the HAdV-55 genome is more similar to the HAdV-14 genome than the HAdV-11 genome, HAdV-55 infection could be associated with severe respiratory infection in certain patients (*17*). Second, the lower levels of herd immunity against HAdV-55 could have an influence on the epidemic of HAdV-55–associated respiratory infection in soldiers in South Korea (*29**,**30*). Severe respiratory illness and outbreaks associated with HAdV-55 in soldiers in South Korea might be similar to those observed in military personnel in China (*13**,**14**,**21**,**22*).

In this study, the proportion of new recruits with HAdV infection among soldiers in South Korea was not as high as expected. More than half of the South Korean soldiers with HAdV infection were on active duty. These findings contrast with previous data in which HAdV-associated respiratory infection has been common among new recruits (*7*). The patients included in this study might have had clinically severe illness rather than mild illness because our institute is the only central referral hospital in the military system in South Korea. Moreover, HAdV easily spreads to advanced training sites or military barracks and it can spread in geographically dispersed military barracks by the movement of soldiers because of prolonged viral shedding (*31*). In this study, some of the patients with HAdV-55 infection who were active duty soldiers were identified in the clusters of patients in 4 military barracks (data not shown). Although the outbreak of HAdV-55 infection was not directly confirmed in this study, outbreaks of HAdV-55–associated respiratory infection might be occurring among active duty soldiers.

Our study has some limitations. First, the study was performed for a relatively short period (16 months). Although the data might be insufficient to reflect the epidemiology of respiratory HAdV infection among military personnel in South Korea, the variation of HAdV type over time and the impact of the emergence of HAdV-55 on clinical severity can be observed. Considering the long-term experience of the US military with respiratory HAdV infection, the distribution of HAdV types might show a substantial difference in the military and community populations in South Korea. Thus, a surveillance system must be established to detect the circulation of the HAdV type among military personnel. Second, the study population included patients who needed hospitalization or an emergency department visit. Among soldiers in South Korea with HAdV infection, patients who had severe clinical signs and symptoms might be those who were primarily enrolled in this study. Nevertheless, our results suggest that of the several HAdV types, HAdV-55 and HAdV-4 might be implicated in HAdV respiratory infections among soldiers in South Korea. Furthermore, active duty soldiers and new recruits could have a substantial disease burden caused by respiratory HAdV infection. Third, we could not determine HAdV types in 11 cases. The viral titer in the clinical specimen might have been low and the viral DNA degraded because some respiratory specimens were not frozen until the molecular experiment. Using the 2-step hemi-nested PCR, we did not observe a DNA band in 11 samples.

This study is important because it is a prospective study on patients with AFRI. Studies on the HAdV type in soldiers in South Korea since 2012 are limited. However, the previous studies provided data on the epidemiology of the HAdV type in patients with severe clinical manifestations or in those with HAdV respiratory infection. These conditions might result in bias in the epidemiology of HAdV infections among military personnel in South Korea. 

In conclusion, our study found that HAdV was the most prevalent virus among soldiers with AFRI in South Korea. In particular, HAdV-55 and HAdV-4 were the prevalent types in soldiers with HAdV-associated respiratory infection. HAdV-55 was associated with severe clinical outcomes. Further studies are needed to verify which HAdV types are associated with AFRI in military recruits. In addition, studies on the introduction or development of an effective vaccine against HAdV-55 and HAdV-4 should be considered.
